# Chemistry of Hydroxypropyl Cellulose Oxidized by Two Selective Oxidants

**DOI:** 10.3390/polym15193930

**Published:** 2023-09-28

**Authors:** Raluca Ioana Baron, Gabriela Biliuta, Ana-Maria Macsim, Maria Valentina Dinu, Sergiu Coseri

**Affiliations:** “Petru Poni” Institute of Macromolecular Chemistry of Romanian Academy, 41 A Gr. Ghica Voda Alley, 700487 Iasi, Romania; biliuta.gabriela@icmpp.ro (G.B.); vdinu@icmpp.ro (M.V.D.)

**Keywords:** hydroxypropyl cellulose, NaIO_4_-oxidation, TEMPO-mediated oxidation

## Abstract

Along with the increased usage of cellulose in the manufacture of novel materials, those of its derivatives that have good solubility in water or organic solvents have become increasingly important. In this study, hydroxypropyl cellulose (HPC), a cellulosic derivative with distinct features, was utilized to investigate how two of the most-selective oxidation methods currently available in the literature act on the constituent OH groups of both the side chain and the anhydroglycosidic unit in HPC. The oxidation reactions were carried out first using TEMPO, sodium hypochlorite, and sodium bromide, then sodium periodate (NaIO_4_), for 5 h. A combination of these two protocols was applied. The amount of aldehyde and number of carboxylic groups introduced after oxidation was determined, while the changes in the morphological features of oxidized HPC were, additionally, assessed. Furthermore, utilizing Fourier-transform infrared spectra, X-ray diffraction, and thermogravimetric studies, the chemical structure, crystallinity, and thermal stability of the oxidized HPC samples were examined and compared.

## 1. Introduction

Polysaccharides are nowadays valuable resources for various scientific, industrial, and technological applications, and they are gaining increased attention due to their many advantages over synthetic polymers. The shift towards using polysaccharides in scientific research and industrial applications, while gradually replacing synthetic polymers, can be attributed to their unique and intrinsic features such as biocompatibility, biodegradability, bioactivity and availability. Last but not least, the growing pressure from society to eradicate the problems related to environmental pollution and degradation have contributed essentially to this transition [1,2,3,4]. Among polysaccharides, cellulose is renewable, cheap, and, most importantly, biodegradable, making it the most plentiful polysaccharide on Earth [5]. Due to strong intramolecular and intermolecular bonds, cellulose is not soluble in water or most organic solvents [6]. To address this critical issue, water-soluble cellulose derivatives are frequently selected, particularly in medical or technical applications. One of the best examples is hydroxypropyl cellulose (HPC), a cellulose derivative with good solubility in water and organic solvents and good chemical stability and processability [7,8]. HPC is obtained through the partial etherification of cellulose with hydroxypropyl groups [9], having *β-D*-glucose units in which the free hydroxyl groups are partially or completely substituted by hydroxypropyl groups. HPC exhibits liquid crystallinity in aqueous solution and is stable and safe enough to be utilized as an excipient in pharmaceutical tablets. As a result, it can be used to build functional materials [10,11,12]. In order to use HPC in a wide range of applications, it is important to introduce reactive groups that allow covalent cross-linking. To this purpose, we have focused on the introduction of reactive carbonyl and/or carboxyl groups through the oxidation of HPC using selective oxidizing agents. Thus, fundamental research into HPC is a worthwhile endeavor. The oxidation reaction, in particular, adds value to polysaccharides by introducing new functionalities into their structure, such as ketone, aldehyde, or carboxyl, or even a combination of these, with significant benefits for subsequent functionalization and the modification of physicochemical properties. Several approaches for HPC oxidation agents have been reported lately. Edgar et al. reported the oxidation of HPC on the 2-hydroxypropyl side chain to acetonyl moieties in the presence of sodium hypochlorite to produce hydrogels with self-healing properties [10]. Recently, Seelinger et al. used a TEMPO-mediated oxidation protocol to oxidize secondary hydroxyl groups of HPC to carbonyl groups under mild conditions, with a controlled degree of oxidation [13].

In our work, we presumed that a comprehensive examination of the oxidation of HPC in the presence of the most selective reagents reported in the literature, i.e., TEMPO radical and NaIO_4_, could provide fresh insights into the behavior of HPC in such reactions. This approach becomes even more appealing since, to the best of our knowledge, no research has been carried out on HPC oxidation with NaIO_4_, let alone in a mixed system with TEMPO and NaIO_4_.

TEMPO is a stable nitroxyl radical, water soluble, and widely used in the presence of NaClO and NaBr for the selective oxidation of its primary –OH groups to –COOH groups, forming intermediate aldehyde groups [14,15,16,17,18,19,20,21,22,23]. On the other hand, NaIO_4_ is an efficient reagent used for the selective oxidation of secondary hydroxyl groups in polysaccharides to aldehyde groups, with the simultaneous cleavage of the C2–C3 linkage [24]. These two selective oxidation methods can be combined in a one-pot reaction to obtain highly carboxylated polysaccharides simultaneously, at the C6 but also at C2 and C3 positions.

## 2. Materials and Methods

### 2.1. Materials

Hydroxypropyl cellulose (HPC, average Mw~80,000, average Mn~10,000), 2,2,6,6-tetramethyl-1-piperidine-1-oxyl radical (TEMPO), sodium bromide (NaBr), sodium periodate (NaIO_4_), 15% sodium hypochlorite (NaClO) and other chemicals and solvents were of pure grade (Sigma Aldrich, St. Louis, MO, USA), and were used without further purification.

### 2.2. Preparation of Oxidized HPC

(i) TEMPO-mediated oxidation of HPC (HPC_T): HPC (2.5 g) was dissolved in 70 mL of distilled water under vigorous stirring. TEMPO (0.2 mmol/g HPC) and NaBr (2 mmol/g HPC) were then introduced into the reaction mixture. Subsequently, a NaClO solution (ca. 15% active chlorine, 20 mmol/g HPC) was added to the reaction mixture while stirring continuously. The pH of the solution was carefully maintained at about pH 10 by adding 2 M NaOH solution. The reaction was carried out for 5 h at room temperature (RT), after which it was quenched by adding a few drops of ethanol.

(ii) NaIO_4_ oxidation of HPC (HPC_P): HPC (2.5 g) was dissolved in 70 mL of distilled water with vigorous stirring, and then NaIO_4_ (4 mmol/g of HPC) was added to the HPC solution. The reaction was carried out at RT in the dark for 5 h. Throughout the reaction, the pH of the suspension was approximately 4. After 5 h, the excess of periodate was decomposed with ethylene glycol.

(iii) Combined TEMPO–periodate oxidation of HPC (HPC_TP): this oxidation protocol was developed in our group previously, to prepare tricarboxicellulose [25]. HPC (2.5 g) was dissolved in 70 mL of distilled water under vigorous stirring. TEMPO (0.2 mmol/g HPC), NaIO_4_ (4 mmol/g HPC), NaBr (2 mmol/g HPC), and NaClO solution (20 mmol/g HPC) were then added. The flask was covered with aluminum foil to prevent the decomposition of periodate under the action of light. After five hours, the reaction was stopped, and the pH was raised to 7.

All reaction products were dialyzed (molecular-weight cut-off (MWCO)—3500 Da) against distilled water for five days, and then dried using lyophilization.

### 2.3. Characterization of Oxidized HPC

#### 2.3.1. The Fourier-Transform Infrared (FTIR) Measurements

Fourier-transform infrared (FTIR) spectra were acquired using an IRAffinity-1S spectrometer (manufactured by Shimadzu Corp., Kyoto, Japan) in conjunction with customized infrared (IR) software (LabSolution IR Version 2.27) developed by LabSolutions (Shimadzu Corp., Kyoto, Japan). Scanning was performed with a resolution of 4 cm^−1^ and the range covered was from 4000 cm^−1^ to 500 cm^−1^. The spectra of all samples were collected using transmission-mode scanning. A pellet containing 200 mg potassium bromide and 1 mg sample was prepared by pressing the sample into the pellet.

#### 2.3.2. Nuclear Magnetic Resonance Spectroscopy (NMR)

Proton and carbon NMR experiments were recorded with a Bruker Avance Neo 400 MHz instrument (Bruker BioSpin, Rheinstetten, Germany) operating at 400.1 MHz for ^1^H and 100.6 MHz for ^13^C. The samples were solubilized in D_2_O at RT, transferred to 5 mm Wilmad 507 NMR tubes and recorded with a 5 mm direct detection probe with four nuclei (H, C, Si, F). Chemical shifts are given in δ units (ppm). All spectra were processed using the program TopSpin 2.1 from BRUKER.

The ^1^H-NMR technique was used to determine the molar substitution (*MS*) of hydroxypropyl cellulose samples. The *MS* was calculated using Equation (1) [26]:

(1) $$MS=\frac{7A}{3\left(B-A\right)}$$

where *A* represents the integral of methyl protons (0.47–1.5 ppm), and *B* represents the integral of the cellulose backbone together with CH_2_ and CH protons from hydroxypropyl (2.6–4.6 ppm).

The degree of substitution (*DS*) of the hydroxypropyl group in hydroxypropyl cellulose (HPC) samples can be determined from the ^1^H-NMR and ^13^C-NMR spectra. Knowing the ratio *R* = number of inside CH_3_(I_2_)/number of terminal CH_3_(I_1_), from the ^13^C NMR spectrum, and the *MS* value from the ^1^H-NMR spectrum, we calculated the value of *DS* using Equation (2) [26]:

(2) $$DS=\frac{MS}{1+R}$$

where *R* = *I*_2_/*I*_1._

The relationship between the molar substitution (MS) and the content of the hydroxypropyl group (HC) is given by Equation (3):

(3) $$HC=\frac{58MS}{162+58MS}\times 100$$

where the molar weight of hydroxypropyl is 58, and the value of 162 represents the molar weight of the anhydroglucose (AGU) unit [27].

#### 2.3.3. Environmental Scanning Electron Microscopy (SEM)

For preparing the samples for SEM, as films, solutions of each sample were dried at 40 °C in a PFA Petri dish. Then, samples were frozen in liquid nitrogen, fractured and analyzed in cross-sections. The morphology of unmodified and oxidized HPC was investigated using a Verios G4 UC scanning electron microscope (SEM) from Thermo Scientific, Brno, Czech Republic. SEM studies were performed in a high-vacuum mode using a secondary electron detector (Everhart–Thornley detector, ETD, FEI Company, Hillsboro, OR, USA) at an accelerating voltage of 5 kV.

#### 2.3.4. Crystallinity Determination using X-ray Diffraction (XRD)

Using a Rigaku Miniflex 600 diffractometer, an X-ray diffraction investigation was carried out utilizing CuK-emission in the angular range 2–50° (2) with a scanning step of 0.01° and a recording rate of 2°/min. Applying the peak height technique [28] and Equation (4), the crystallinity index (*Cr.I.*) was calculated.

(4) $$Cr.I.=\frac{I_{002}-I_{Am}}{I_{002}}\times 100$$

#### 2.3.5. Zeta Potential

The ζ-potential was measured at 25 °C using laser Doppler electrophoresis equipment (Malvern Nano-Zetasized ZS, Malvern, UK). The samples were diluted to include 0.1 weight percent solids. Each measurement was repeated three times. For kα >> 1 (k—Debye–Hűckel parameter and α—particle radius), the Smoluchowski relationship was applied, as shown in Equation (5):

ζ = *ηµ*/*ε* (5)

where *η* is the viscosity, *µ* is the electrophoretic mobility and *ε* is the dielectric constant.

#### 2.3.6. Determination of Mass Yield

The mass yield of HPC samples was calculated according to the method reported by Chen et al. [29]. The determination of the weight ratio between modified HPC and pristine HPC was carried out using Equation (6).

(6) $$mass\;yield\;\%=\frac{m_{2}}{m_{1}}\times 100$$

where *m*_1_ is the amount of HPC used (mg), and *m*_2_ is the amount of modified HPC obtained (mg). Determinations were made in triplicate.

#### 2.3.7. Conductometric and Potentiometric Titration

##### Determination of Carboxyl-Group Content in HPC_T and HPC_TP

Conductometric titration was used to determine the number of carboxyl groups present in the oxidized HPC samples [30,31]. A weight of 0.05 g of oxidized HPC was dispersed in 30 mL of deionized water. Then, 1.5 mL NaCl solution (0.01 M) was added to the HPC suspension. The pH of the solution was adjusted to 2.5–3.0 by adding 0.1 M HCl. The conductometric titration was performed using a TitroLine^®^ 5000 (Radiometer, Copenhagen, Denmark) from SI Analytics and a Metrohm 914 pH/conductometer from Switzerland, with 0.1 M NaOH as the titration solution. Using Equation (7), we could calculate the number of carboxyl groups:

(7) $$carboxyl\;content\;\left(\frac{mmol}{g}\right)=\frac{\left(V_{2}-V_{1}\right)C}{m}$$

where *V*_2_ and *V*_1_ represent the titration curve’s inflexion points and represent the quantity of NaOH consumed, ml; *C* was the NaOH concentration, mol/L; and *m* was the weight of dried HPC, g.

##### Determination of Aldehyde-Group Content in HPC_P

The alkali consumption method was applied to determine the aldehyde-group concentration of HPC_P. (More details are included in Supplementary Information). The Canizzaro reaction of HPC_P proceeded rapidly with a stoichiometric consumption of hydroxyl ions per dialdehyde group. After precisely adding HPC_P (0.10 g) and NaOH solution (5.00 mL, 0.1 M) into an Erlenmeyer flask with a volume of 100 mL, the flask was then stirred with a magnetic stirrer for a period of thirty minutes in order to dissolve the sample. Following that, a precise amount of HCl solution (7.50 mL) with a concentration of 0.1 M was added, and then 15.00 mL of distilled water was added. The titration to the end point was conducted with a solution of 0.1 M NaOH after 5 drops of phenolphthalein indicator were added. The dialdehyde concentration in the sample was calculated from Equation (8):

(8) aldehyde group content=C1V1−C2V2m162×1000×100%

where *C*_1_ (mol/L) represents the concentration of the NaOH solution, *V*_1_ (mL) represents the volume of the NaOH solution, *C*_2_ (mol/L) represents the concentration of the HCl solution, *V*_2_ (mL) represents the volume of the HCl solution, *m* (g) represents the mass of the dried HPC_P, and 162 represents the molecular weight of one repeated unit of anhydroglucose.

#### 2.3.8. Thermogravimetric Measurements

Thermogravimetric measurements were taken with a STA 449F1 JUPITER instrument, manufactured by Netzsch GmbH in Selb, Germany. Calibrations of temperature and sensitivity were conducted with indium over a temperature range of 30–700 °C. After the weighing of the solid samples, which ranged from around 5.0 to 11 mg, the samples were placed in alumina pans to be subjected to TG measurements. The temperature range employed was between 30 and 700 °C, and the heating rate was 10 °C/min. This was performed in an environment of dry nitrogen with a flow rate of 50 mL/min. The NETZSCH PROTEUS 4.2 software (Netzsch, GmbH, Selb, Germany) was used to perform the analysis of the data.

## 3. Results and Discussion

### 3.1. Mechanism of Reaction

**Figure 1:**
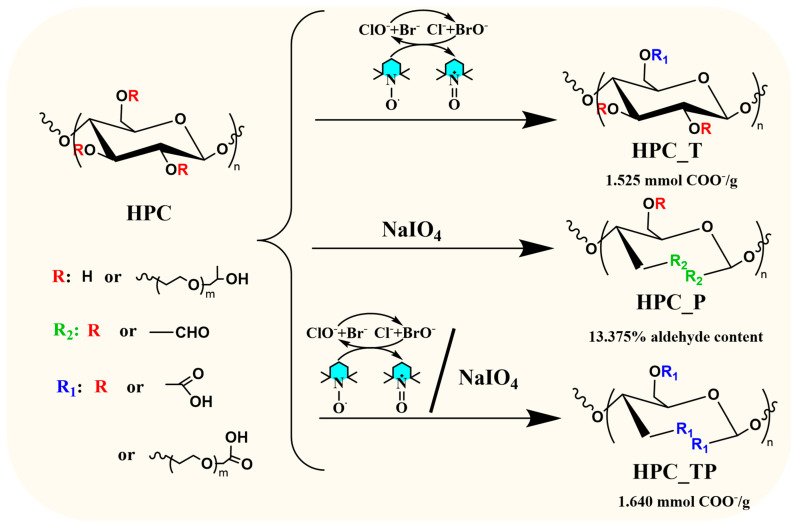
Schematic representation of HPC oxidation of in the presence of different oxidizing agents.

In recent years, several in-depth studies have been reported on the role of polysaccharide-specific oxidizing agents in general and of cellulose [32,33] in particular, due to the proliferation of natural polymer-based hydrogels, in which cellulose oxidation products play the key role. The stable TEMPO radical and its derivatives, *N*-hydroxyphtahlimide (NHPI) and the non-persistent phthalimide-*N*-oxy radical (PINO) [34,35], as well as NaIO_4,_ have been extensively used for the selective oxidation of polysaccharides. These oxidation protocols are among the most selective in this type of reaction. Another useful oxidation method capable of producing 2,3,6-tricarboxy compounds in a single step is the one that combines the TEMPO radical with NaIO_4_. Figure 1 illustrates how the oxidation of HPC could proceed in the presence of all three proposed protocols.

According to Figure 1, the TEMPO/NaBr/NaClO oxidation system enables the selective conversion of primary hydroxyl/propyl groups to carboxyl groups. In this system, sodium hypochlorite plays a predominant role as the primary oxidant, oxidizing the TEMPO radical to the *N*-oxoammonium ion TEMPO^+^, which converts the primary hydroxyl groups to carboxyl groups. On the other hand, NaIO_4_ oxidizes the secondary hydroxyl groups to introduce dialdehyde groups into the structure of HPC, leaving the primary groups unaffected. Oxidation in the presence of periodate is a highly selective reaction, the hydroxyl groups from C2 and C3 position being converted to aldehyde by cleaving C2-C3 bonds [36]. When combining the two above oxidation protocols, from a theoretical perspective, it becomes plausible that both the OH groups from the side chain and the OH groups from the anhydroglycosidic unit (the unsubstituted ones) could be oxidized. The sample oxidized in the presence of TEMPO radical gave the best yield (η = 95%). When oxidation is carried out in the presence of NaIO_4_, more side reactions and degradation take place and the yield obtained is lower (η = 88%), the smallest being in the case of the reaction with TEMPO and NaIO_4_ (η = 70%).

Furthermore, when analyzing the results obtained from the oxidation process and quantifying the nature of the functional groups obtained, we observed that during the oxidation process with the TEMPO radical, the ketone groups could also be obtained, which were formed when oxidation took place at the hydroxyl groups in the side chain of the HPC (see the ^13^C-NMR results). Similar results have been reported in previous works [37,38,39]. Summarizing, we can observe that depending on the nature of the substituents in the C2, C3, and C6 positions (hydroxyl or propyl), the carboxyl, aldehyde, and ketone groups can all be obtained as follows:

(i) When the oxidation takes place in the presence of the TEMPO radical, both carboxylic and ketone groups are obtained at the C6 atom;

(ii) When the oxidation takes place in the presence of NaIO_4_, both aldehyde and ketone groups are obtained; 

(iii) when the oxidation takes place in the presence of the TEMPO/NaIO_4_ system, carboxylic, aldehyde, and ketone groups are obtained.

### 3.2. FTIR

**Figure 2:**
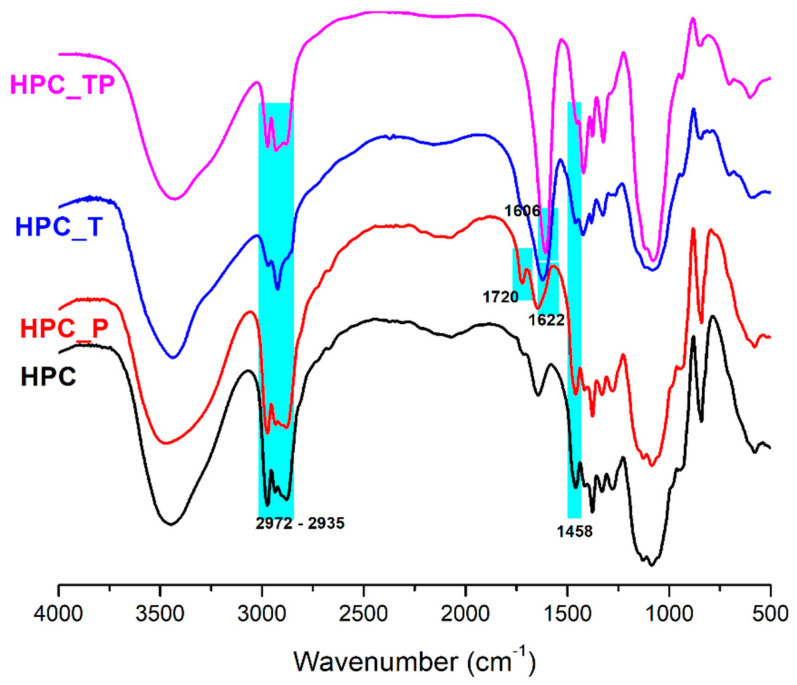
The FTIR spectrum of HPC and the oxidized samples.

To confirm the oxidation reactions of HPC, the FTIR spectra of the samples in the 4000 to 500 cm^−1^ wavenumber regions were registered (Figure 2). The FTIR spectrum of HPC showed two significant bands at 3445 cm^−1^ and 1085 cm^−1^, and shoulders at 2972 cm^−1^ and 2880 cm^−1^; thus, the absorption band range from 3700 cm^−1^ to 3200 cm^−1^ was correlated with the extension vibration of the hydroxyl group (OH-side-chain stretching and OH-ring stretching). The band from 3000 cm^−1^ to 2800 cm^−1^ was associated with the symmetric and asymmetric extension vibrations of the –CH_3_ and –CH_2_ groups (2972 (CH_3_) asymmetric stretching, 2935 (CH_2_) asymmetric stretching, 2900 (CH_2_) symmetric stretching, 2879 (CH_3_) symmetric stretching, and (CH) ring stretching). The OH bending of adsorbed water (hydrophilic hydroxyl group in HPC) was assigned to the characteristic band at about 1643 cm^−1^. In the fingerprint region, which includes wave numbers between 1500 and 900 cm^−1^, there were many bands and shoulders; thus, 1458 cm^−1^ was assigned to O-H, C-H bending, and –CH_2_ deformation; 1377 and 1327 cm^−1^ were due to –CH_2_ wagging and C-H and O-H bending vibration modes, and 1278 cm^−1^ was assigned to O-H bending and C-H wagging. The bands at 1128, 1053, and 1085 cm^−1^ were assigned to C-O stretching vibration [40,41]. The peak at 943 cm^−1^ represents the in-phase vibrations from propyl linkages and appeared as a weaker band attached to the band at 1053 cm^−1^. The FTIR absorption band appearing at 841 cm^−1^ was due to C-O deformation and –CH_2_ rocking, and it is defined as an “amorphous” absorption band.

Comparing the spectra for HPC and oxidized HPC_P, it was observed that in the FTIR spectra of HPC_P, a novel strong C=O stretching vibration at 1720 cm^−1^ appeared. It is noted that this vibration was absent from the spectrum of the HPC [36]. Also, the center of gravity of the (OH) band had a very small blue shift, from 3444 to 3464 cm^−1^, this shift being caused by the (OH) groups from HPC_P and less by changes in the strength of the hydrogen bonds between molecules. No other significant changes were observed in comparison to the original HPC sample. In the FTIR spectra of HPC_T and HPC_TP, a new band at 1606 cm^−1^ for HPC_TP and 1622 cm^−1^ for HPC_T appeared [42], this band being assigned to the carboxyl groups in anionic form (νCOO^-^), indicating oxidation of the starting product, in positions C-2,3,6 (HPC_TP) and C6 (HPC_T). Another aspect to highlight is the fact that the intensity of the symmetrical stretching of the methyl and hydroxypropyl groups from 2972 cm^−1^ and 2935 cm^−1^ was less intense and slightly shifted towards lower frequencies, indicating that the C-H groups (in C6, C2 and C3) present in HPC were destroyed by oxidation. In addition, the absorption band at 1458 cm^−1^,associated with the size of the crystalline structure of the cellulose, became less prominent in samples HPC_T and HPC_TP, which means that a decrease in its intensity implies a decrease in the degree of crystallinity of the samples. In addition, the intensity of this band was reduced along with the conversion of OH into COO^−^ groups.

FTIR spectroscopy can also be used to determine the crystallinity of a wide variety of materials [43]. In order to perform this, a metric known as the “*total crystalline index*” (TCI) was developed. This metric is calculated by taking the ratio of the intensities of the peaks occurring at 1377 cm^−1^ and 2879 cm^−1^ [44,45]. This parameter is closely related to the “*lateral order index*” (LOI), which is determined by considering the ratio of peak intensities from 1413 cm^−1^. The peak at 1413 cm^−1^ is often assigned to the crystalline cellulose I, while the peak at 943 cm^−1^ is characteristic of the amorphous component of cellulose [46]. As a result, the LOI provides insight into the overall crystallinity of the sample, with the LOI value increasing as the crystallinity decreases [47]. The intensity of hydrogen bonding, or in the other words, the degree of intermolecular regularity of the cellulose network, is defined by another parameter, known as the “*hydrogen bond index*” (HBI). The degree to which crystalline and amorphous structures coexist in cellulose derivatives is an important factor in determining both the supramolecular structure of the derivatives and the quality of the cellulose fibers they produce [48]. The hydrogen bonding intensity (HBI) may be determined from the FTIR spectra by comparing the heights of the bands at 3444 cm^−1^ and 1321 cm^−1^ and using the ratio of the two values [49]. High HBI values are often caused by the fibers having a greater cellulose crystallinity, according to *Poletto* et al. [50]. By using FTIR spectroscopy, one is able to easily and reliably determine the crystallinity indices (LOI and TCI) as well as the HBI. Their values are presented in Table 1. Considering the LOI values for the oxidized hydroxypropyl cellulose samples, there was a significant decrease in the degree of crystallinity for the HPC_T and HPC_TP samples. This finding suggests that after oxidation, crystalline domains are unevenly distributed throughout the cellulose samples. A laterally organized cellulose structure is associated with a high LOI value, which was observed for the samples HPC_T and HPC_TP. The highest values of the TCI index and, at the same time, low LOI values for pure HPC and HPC_P samples, shows that these samples have a higher cellulose crystallinity and can present more amorphous domains in the cellulose structure [50].

### 3.3. NMR

**Figure 3:**
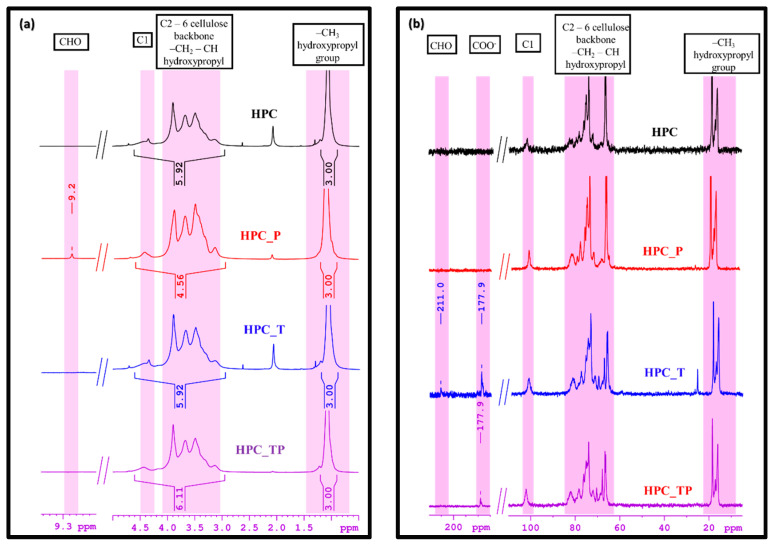
(**a**) ^1^H-NMR spectra of pristine HPC and oxidized samples (HPC_T, HPC_P and HPC_TP), and (**b**) ^13^C-NMR spectra of HPC and oxidized samples (HPC_T, HPC_P and HPC_TP), in D_2_O.

The ^1^H-NMR spectra for the HPC samples showed a characteristic peak at 4.44 ppm for anomeric protons H1 and in the region of 3.15–3.89 ppm for the rest of the H2–H6 protons for the glucose unit. The peaks for the methylene and methyl groups of the 2-hydroxypropyl substituent overlapped with those of the glucose moiety in the region of 3.15–3.89 ppm. The methyl protons of the 2-hydroxypropyl substituent exhibited resonance signals at 1.09 ppm (Figure 3a). After oxidation with NaIO_4_, a new signal was observed to have developed in the ^1^H-NMR spectrum of the HPC_P sample. This signal is thought to be associated with aldehyde CHO groups, which can be seen as proof that the reaction proceeded as expected.

The ^13^C-NMR spectra exhibited the signals of carbon atoms characteristic of the cellulose backbone. Thus, the C1 atom appeared at 102 ppm, while the signals in the region of 83–65 ppm were attributed to the carbon atoms CH_2_-CH of the hydroxypropyl group and C2–C6 carbon atoms of the cellulose backbone. According to the literature [51], CH_3_ carbon from internal hydroxypropyl groups resonates at 16 ppm, whereas CH_3_ carbon from external hydroxypropyl groups resonates at 18 ppm. In Figure 3b, where the ^13^C-NMR spectra of HPC and oxidized samples are presented, we can observe two new peaks, at 177.9 ppm and at 211 ppm, for the new products obtained. So, the peak from 177.9 ppm can be assigned to the carbonyl carbons presented in the oxidized samples. The other one, at 211 ppm, is attributed to ketonic groups present, probably because of the NaClO solution assisting in the reaction.

The values of the molar substitution were 2.25 and 2.34 for the HPC_TP and HPC_T samples, respectively (Table 2), indicating a stronger oxidation of these samples compared to the HPC_P, where the MS was 4.49. The percentage of the hydroxypropyl group (HC) content was the lowest value for the HPC_T and HPC_TP samples, which were oxidized at carbon atoms C6 and C2, 3 and 6. The DS value decreased with the number of the hydroxypropyl-group equivalents. Also, after oxidation, we observed a drastic decrease in the hydroxypropyl content in the HPC matrix, with the value of DS decreasing from 2.94 in HPC to 0.97 in the HPC_PT sample.

### 3.4. SEM

**Figure 4:**
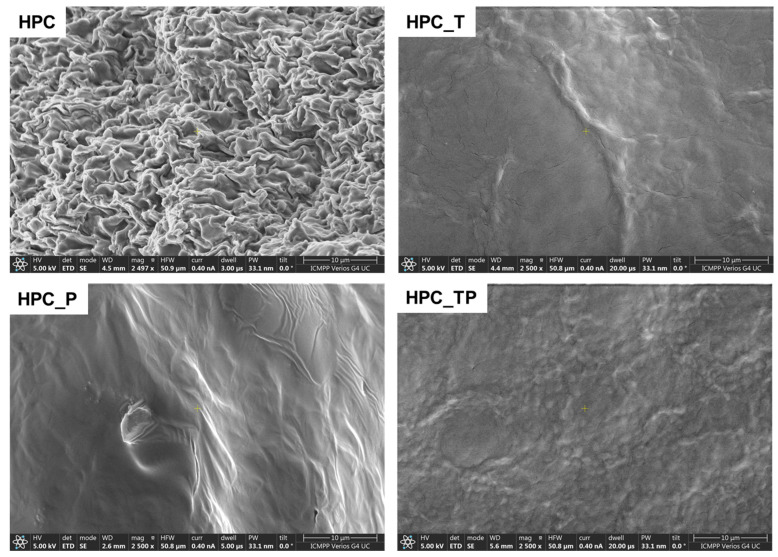
SEM images of HPC and oxidized HPC in the presence of the TEMPO/NaClO/NaBr system (HPC_T); in the presence of NaIO_4_ (HPC_P); and in the presence of the TEMPO/NaClO/NaBr/NaIO_4_ system (HPC_TP).

The morphology of the unmodified HPC and oxidized HPC, obtained in the form of films using the solvent-casting method, was examined using SEM (Figure 4).

In the unmodified HPC samples, an advanced structuring appeared, with a cauliflower-like appearance (Figure 4) [52]. As the macromolecular chains are less mobile in the crystalline structure, they might have a tendency to aggregate or form specific patterns during the formation process or while the material is solidifying [53]. This aggregation could give rise to the cauliflower-like morphology observed for the unmodified HPC films. It can be seen that after oxidation, the samples no longer show their initial morphology, thus losing the fibrous structure of the HPC itself. In contrast, in the oxidized samples, these well-defined domains can be partially interrupted by the formation of a significant amount of amorphous regions due to the oxidation process.

### 3.5. XRD

**Figure 5:**
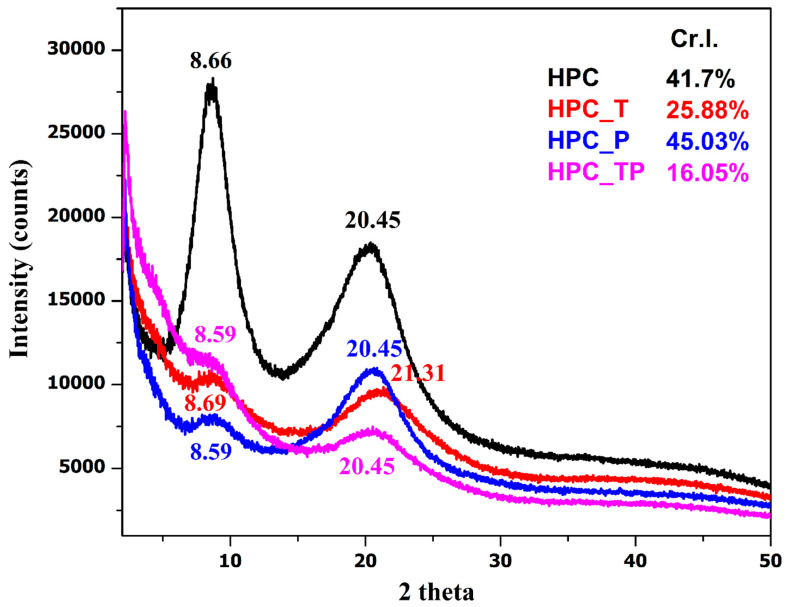
The XRD diffractograms of the analyzed samples and their crystallinity indices.

For comparison, the X-ray diffractograms of the original HPC and those after various oxidation reactions are shown in Figure 5. XRD patterns show that the crystallinity of the HPC was reduced as a result of the progressive breaking of the pyranose rings naturally present in the molecule, except for the HPC_P sample.

XRD patterns reveal that the crystallinity of oxidized HPC undergoes a dramatic reduction as a result of the progressive breakage of the pyranose rings that are naturally present in the molecule [54]. The distinctive two signals of HPC could be seen in each X-ray diffractogram of the samples. These signals were centered at 2θ = 8.60° for the crystalline component, and the ordered amorphous fraction of HPC was centered at 2θ = 20.45° [55,56]. In the case of the oxidized samples with carboxylate groups (HPC_T and HPC_PT), the lowering of the crystallinity index (Cr.I.) value was, without doubt, the result of the oxidation process. This finding suggests that the newly formed carboxylate groups are present not only on the surfaces of the oxidized samples but also, most likely, in some of the internal crystallites [57]. In the case of the sample oxidized with periodate HPC_P, an increase in crystallinity was observed. This increase is attributed to the increased reactivity of periodate; during the oxidation process the hydroxyl groups present in the amorphous region are oxidized to aldehyde groups, groups that lead to the depolymerization of HPC. Similar results were reported by Mendoza et al., who showed that although the periodate concentration had a significant effect on crystallinity, it did not affect the cellulose crystal size [23].

### 3.6. The Zeta Potential and the Quantity of the Carboxyl Content

The zeta potential is a measure of the electrokinetic features that occurred at the interface between the solid and the liquid, as well as the interactions that rose between them. High levels of chemical and electrochemical interactions may be seen at the interface between a solid and a liquid; these interactions are fundamentally distinct from those that are present in the solid phase. Because of these two characteristics, it is possible to ascertain whether or not there is a net charge present at the boundary between the two phases of the system: (i) the processes of the dissociation of the surface-charged groups, and (ii) the adsorption of potential by the ions from a solution [58]. The zeta potentials of the samples were determined in deionized water at a concentration of 1.0 mg⋅mL^−1^, and the results are shown in Table 3. From the table, it can be seen that the zeta potential values of HPC were negative, which means that the stability of HPC in aqueous solutions is high. The zeta potential values of the oxidized HPC were also more negative than those of the HPC, ranging from −14.5 mV for the HPC_P to −26.3 mV for the HPC_TP. The HPC_T sample had a ζ potential value of −24.4 mV, indicating that the significant repulsive force developed between the groups of carboxylated molecules, introduced after oxidation with TEMPO, had the highest zeta potential value, and provides a stable, improved solution. In the case of the HPC_TP sample, the value of the ζ potential was only −26.3 mV, despite the fact that it contained a larger total number of carboxyl groups. This discrepancy can be explained by the fact that the cellulose chains were broken apart.

Table 3 shows the content of the carboxylate obtained after oxidation. The oxidation of HPC in a typical TEMPO-mediated oxidation formed a highly carboxylated fraction (1.525 mmol COO^−^/g). The introduction of sodium periodate into this system increased the carboxylate content to 1.640 mmol COO^−^/g. Since sodium periodate adds additional sites (i.e., aldehyde groups) for carboxylated synthesis, this increase in carboxylate concentration was anticipated. In hydroxypropyl cellulose, the periodate oxidation process did not considerably introduce carboxylate groups (0.05 mmol COO^−^/g), which was to be expected.

### 3.7. Thermal Stability

**Figure 6:**
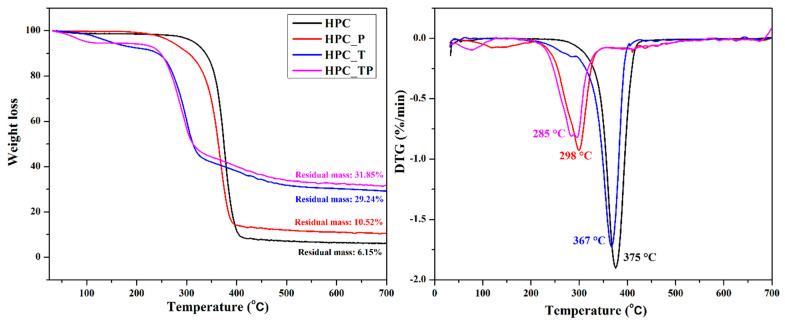
Thermogravimetric (TG) and the corresponding derivative thermogravimetric (DTG) curves of pristine hydroxypropyl cellulose (HPC) and the oxidized samples (HPC_T, HPC_P and HPC_TP).

Thermogravimetric analysis, more often known as TGA, is one of the most prevalent procedures for characterizing materials using thermal techniques. This provides quantifiable information on the change in weight loss of a sample in response to temperature changes. To evaluate the differences between thermograms, the technique of differential thermal analysis is used. Figure 6 depicts the usual thermogravimetric (TG) curve and its associated derivative thermogravimetric (DTG) curve of HPC, as well as the oxidized samples (HPC_T, HPC_P, and HPC_TP). As can be seen in the figure, the thermal disintegration of HPC took place through a single-step process, with the highest level of degradation occurring at 356 °C. As is evident from the information shown in Table 4, the initial decomposition temperatures of oxidized hydroxypropyl cellulose were lower than those of the hydroxypropyl cellulose. Specifically, these temperatures were 340 °C for HPC_TP, 312 °C for HPC_T, and 344 °C for HPC_P, and corresponded to the degradation of HPC anhydro-glucuronate units. It was noticed that the temperature at which the substance decomposed had a strong correlation with the amount of crystallinity present.

After heating HPC to 600 °C, a residue of about 6% by weight remained. The thermal stability of HPC was greatly reduced when it underwent oxidation to create HPC_T and HPC_TP, and the process no longer operated in a single step. It is important to point out that the temperatures at which the oxidized compounds HPC_T and HPC_PT were entirely destroyed were noticeably lower than they were in the case of the sample that had not been changed. On the other hand, the temperature of the HPC_P sample was much closer to that of the unmodified HPC (Table 4, Tonset and Tendset). The amount of new groups introduced into the structure of the HPC determined drastic changes in the thermal stability of the sample, both by the number of aldehyde and/or carboxylic groups introduced, and by their position in the structure of the macromolecular chain [59]. The residual weights of the oxidized samples was noticeably larger than that of regular HPC, which can be explained by the existence of residual metal oxide, which was the consequence of the thermal disintegration of COONa groups [60].

## 4. Conclusions

Three possible pathways for the oxidation of HPC were studied in this study, utilizing the most popular oxidation protocols: TEMPO as a mediator (in the presence of NaClO and NaBr) and NaIO_4_. In addition, a hybrid procedure involving a “*one pot*” oxidation process in the presence of both the TEMPO radical and NaIO_4_ was developed. The oxidized product yield was dependent on the oxidation system, as was the carboxylic-group content of the structural unit. Even though the morphology of the oxidation products appears to be unaffected by the oxidative environment, as seen in the SEM images, significant differences between the oxidized samples were observed in terms of their crystallinities and thermal behaviors, involving extensive structural changes that depend on the oxidation system used. Based on these first promising results, our future work will focus more on the development of applications involving oxidized HPC: controlled drug delivery systems, food packaging materials, and sorbents for wastewater remediation.

## Figures and Tables

**Table 1:** Crystallinity indices of HPC and the oxidized samples.

Sample	TCI (A_1377_/A_2879_)	LOI (A_1413_/A_943_)	HBI (A_3444_/A_1328_) (%)
HPC	1.32	0.39	9.2
HPC_T	1.45	1.15	6.6
HPC_P	1.30	0.39	9.3
HPC_TP	1.16	1.18	7.0

**Table 2:** The molar substitution (MS), the degree of substitution (DS) and the content of hydroxypropyl group (HC) of the analyzed samples.

Sample	^a^ MS	^b^ DS	^c^ HC %
HPC	4.93	2.49	63.8
HPC_T	2.40	1.55	45.59
HPC_P	4.49	1.75	46.18
HPC_TP	2.25	0.84	44.61

^a, b, c^ are calculated according to Equations (1)–(3).

**Table 3:** Carboxylate content (mmol/g HPC), aldehyde content (%), mass yield (%) and zeta potential value (mV) of the samples.

Sample	^a^ COO^−^ Content (mmol/g HPC)	^b^ Aldehyde Content (%)	^c^ Mass Yield (%)	^d^ Zeta Potential (ζ, mV)
**HPC**	-	-	-	−8.26
**HPC_T**	1.525	-	95	−24.4
**HPC_P**	0.05	13.375	88	−14.5
**HPC_PT**	1.640	-	70	−26.3

^a^—determined with Equation (7); ^b^—determined with Equation (8); ^c^—determined with Equation (6); ^d^—determined with Equation (5).

**Table 4:** Thermogravimetric data, T onset peak (onset degradation temperature) and T endset peak (final degradation temperature), weight loss (%) and residue values for the analyzed samples (weight remaining at 700 °C).

Sample	Samples Mass (mg)	W%	Total Weight Loss at 700 °C (Residue, %)	T Onset (°C)	T Endset (°C)	T Peak (DTG/°C)
**HPC**	15.42	75.11	6.15	356.8	389.2	375
**HPC_P**	8.23	68.82	29.24	344.6	382.8	298
**HPC_T**	8.36	44.25	10.52	272.3	315.7	367
**HPC_TP**	8.11	45.31	31.85	255.2	311.5	285

## Data Availability

The data presented in this study are available on request from the corresponding authors.

## Supplementary Materials

The following supporting information can be downloaded at: https://www.mdpi.com/article/10.3390/polym15193930/s1. Refs. [61,62,63,64,65] are cited in the Supplementary Materials.
