# Supplementary material for: Chemistry of Hydroxypropyl Cellulose Oxidized by Two Selective Oxidants
Source: Polymers (Basel). 2023 Sep 28;15(19):3930. doi: 10.3390/polym15193930 (PMC10574994; doi:10.3390/polym15193930)
Supplement: Supplementary file 1 [file polymers-15-03930-s001.zip › polymers-2611388-supplementary.pdf]

Supplementary material

Chemistry of Hydroxypropyl Cellulose Oxidized by Two Selective Oxidants

Raluca Ioana Baron *, Gabriela Biliuta, Ana-Maria Macsim, Maria Valentina Dinu and Sergiu Coseri *

"Petru Poni" Institute of Macromolecular Chemistry of Romanian Academy, 41 A Gr. Ghica Voda Alley, 700487 Iasi, Romania

* Correspondence: baron.raluca@icmpp.ro (R.I.B.); coseris@icmpp.ro (S.C.)

Determination of Aldehyde Group Content in HPC_P

Studies of cellulose oxidation in the presence of sodium periodate indicate that dialdehyde cellulose (DAC) is obtained after the oxidation process. The C2-C3 bond of the AGU is selectively cleaved during the periodate oxidation, and two aldehyde groups are created at the specified site via a cyclic iodate diester (see **Figure S1**).

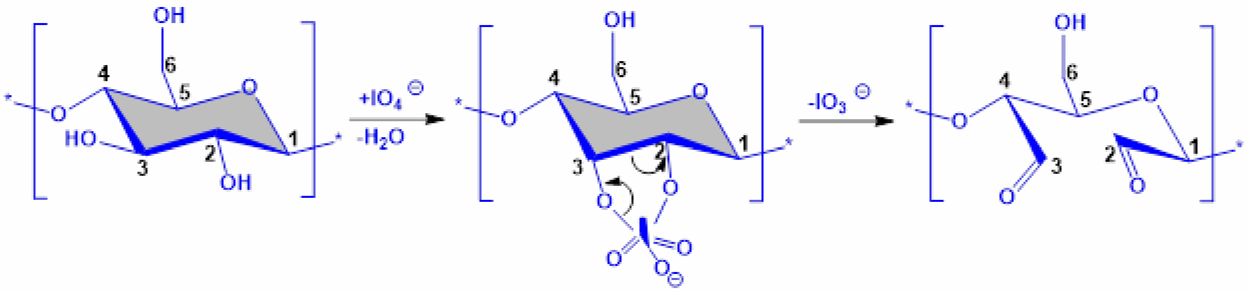

Figure S1. Schematic representation of the formation of DAC by the periodate oxidation of cellulose.

The amount of periodate introduced in the reaction affects the degree of oxidation, i.e., how many of the AGUs are converted into the two aldehyde groups. The aldehyde groups of DAC can form new structures due to their high reactivity; see **Figure S2**. Apart from the free aldehyde (4a), the other four possible structures are intramolecular and intermolecular hemiacetal (S2b and S2e), hemialdol (S2c), and hydrated aldehydes (S2d). The formation of hemiacetal, hydrated aldehyde, and hemialdol are reversible reactions that can lead back to free aldehydes.

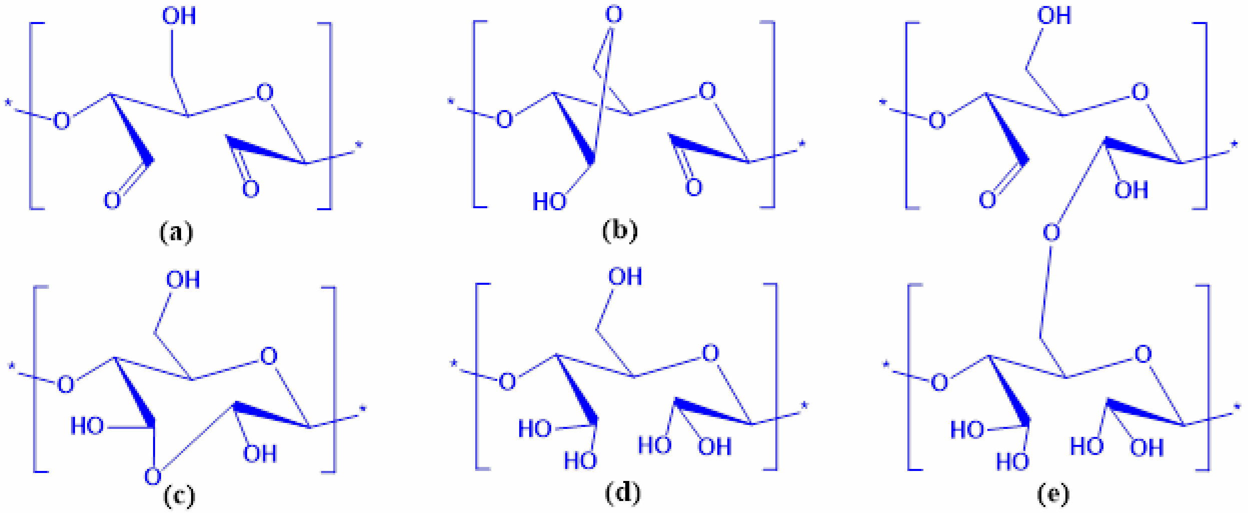

Figure S2. Schematic representation of the possible structures of DAC: (a) free aldehyde, (b) intramolecular hemiacetal, (c) hemialdol, (d) hydrated aldehydes, and (e) intermolecular hemiacetal.

The degree of oxidation, or aldehyde group content, is the number of C2-C3 bonds that have changed into dialdehydes out of the total number of C2-C3 bonds. Various methods have been used to

determine the degree of oxidation, the most commonly used being the reaction with hydroxylamine hydrochloride, $2NH_2OH$-HCl, followed by titration with sodium hydroxide. This is an oxime reaction, in which the hydroxylamine hydrochloride reacts with all of the available aldehyde groups [1,2], see **Figure S3**.

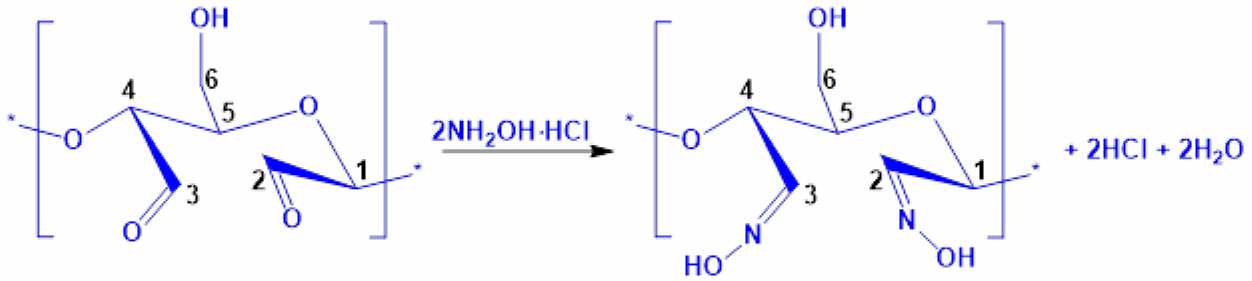

Figure S3. Reaction between dialdehyde cellulose and hydroxylamine hydrochloride.

Another alternative could be to measure the degree of oxidation using an alkaline dissolution technique, in line with the Cannizzaro redox reaction [3]. The amount of introduced aldehyde in our study was determined by consumption of alkali in Cannizzaro reaction [4].

The Cannizzaro reaction of periodate-oxidized HPC proceeds swiftly with a stoichiometric consumption of hydroxyl ions per dialdehyde group. Two potential reaction products (2 or 3) are anticipated from the Cannizzaro reaction of 2,3-dialdehyde HPC; **see Figure S4** [5].

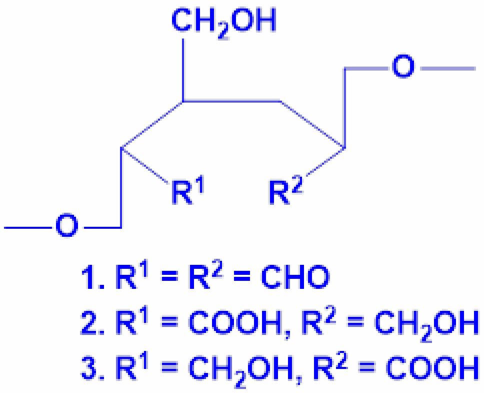

Figure S4. Schematic representation of possible products from the Cannizzaro reaction.

Concluding, in our study, aldehyde group content in HPC_P was determined using the alkali consumption method. Its principle is that double aldehyde can occur in a Cannizzaro reaction in a molecule to determine the dialdehyde content with acid-base titration.

References

1. Sirvio, J.; Hyvakko, U.; Liimatainen, H.; Niinimaki, J.; Hormi, O. Periodate oxidation of cellulose at elevated temperatures using metal salts as cellulose activators, *Carbohydr. Polym.* **2011**, *83*, 1293-1297.
2. Larsson, P.A.; Gimåker, M.; Wågberg, L. The influence of periodate oxidation on the moisture sorptivity and dimensional stability of paper, *Cellulose* **2008**, *15*, 837-847.
3. Wang, X.; Fang, G.; Hu, C.; Du, T. Application of Ultrasonic Waves in Activation of Microcrystalline Cellulose, *J. Appl. Polym. Sci.* **2008**, *109*, 2762-2767.
4. Pommerening, K.; Rein, H.; Bertram, D.; Muller, R. Estimation of dialdehyde groups in 2,3-dialdehyde bead-cellulose, *Carbohydr. Polym.* **1992**, *233*, 219-223.
5. Hofreiter, B.T.; Alexander, B.H.; Wolff J.A. Rapid estimation of dialdehyde content of periodate oxystarch through quantitative alkali consumption, *Anal. Chem.* **1955**, *27*, 1930-1931.
